# Arbuscular mycorrhizal symbiosis and osmotic adjustment in response to NaCl stress: a meta-analysis

**DOI:** 10.3389/fpls.2014.00562

**Published:** 2014-10-17

**Authors:** Robert M. Augé, Heather D. Toler, Arnold M. Saxton

**Affiliations:** ^1^Department of Plant Sciences, University of TennesseeKnoxville, TN, USA; ^2^Department of Animal Sciences, University of TennesseeKnoxville, TN, USA

**Keywords:** arbuscular mycorrhiza, compatible solutes, K^+^/Na^+^ ratio, meta-analysis, NaCl stress, osmotic adjustment, salinity, salt stress

## Abstract

Arbuscular mycorrhizal (AM) symbiosis can enhance plant resistance to NaCl stress in several ways. Two fundamental roles involve osmotic and ionic adjustment. By stimulating accumulation of solutes, the symbiosis can help plants sustain optimal water balance and diminish Na^+^ toxicity. The size of the AM effect on osmolytes has varied widely and is unpredictable. We conducted a meta-analysis to determine the size of the AM effect on 22 plant solute characteristics after exposure to NaCl and to examine how experimental conditions have influenced the AM effect. Viewed across studies, AM symbioses have had marked effects on plant K^+^, increasing root and shoot K^+^ concentrations by an average of 47 and 42%, respectively, and root and shoot K^+^/Na^+^ ratios by 47 and 58%, respectively. Among organic solutes, soluble carbohydrates have been most impacted, with AM-induced increases of 28 and 19% in shoots and roots. The symbiosis has had no consistent effect on several characteristics, including root glycine betaine concentration, root or shoot Cl^−^ concentrations, leaf Ψ_π_, or shoot proline or polyamine concentrations. The AM effect has been very small for shoot Ca^++^ concentration and root concentrations of Na^+^, Mg^++^ and proline. Interpretations about AM-conferred benefits regarding these compounds may be best gauged within the context of the individual studies. Shoot and root K^+^/Na^+^ ratios and root proline concentration showed significant between-study heterogeneity, and we examined nine moderator variables to explore what might explain the differences in mycorrhizal effects on these parameters. Moderators with significant impacts included AM taxa, host type, presence or absence of AM growth promotion, stress severity, and whether NaCl constituted part or all of the experimental saline stress treatment. Meta-regression of shoot K^+^/Na^+^ ratio showed a positive response to root colonization, and root K^+^/Na^+^ ratio a negative response to time of exposure to NaCl.

## Introduction

Osmotic stress caused by salinity and drought remains the most consequential environmental limitation on crop productivity (Boyer, 1982; Zhu, 2003), and the continuing salinization of arable land is projected to greatly increase its negative impacts in the coming decades (Evelin et al., 2009; Porcel et al., 2012). Soil salinity disrupts plant physiological processes by diminishing nutrient and water uptake (Ruíz-Lozano et al., 2012; Hajiboland, 2013) and through toxic ion effects on organelles and enzyme activities (Munns et al., 2006). Physiological mechanisms evolved by plants to deal with salinity stress include accumulation of compatible osmolytes, ion homeostasis, regulation of water uptake by aquaporins, and increased production of antioxidants (Ruíz-Lozano et al., 2012).

Arbuscular mycorrhizal (AM) symbiosis can bolster each of these physiological mechanisms for coping with salt stress (Evelin et al., 2009; Ruíz-Lozano et al., 2012). Relative to nonmycorrhizal (NM) plants, AM plants have been reported to accumulate more proline and other organic solutes (Aggarwal et al., 2012), maintain more favorable K^+^/Na^+^ ratios (Hajiboland, 2013), increase root water transport (Ruíz-Lozano and Aroca, 2010a; Aroca et al., 2012) and improve antioxidant capacity (Ruíz-Lozano et al., 2012). However, while considerable research has documented the role of AM fungi in promoting plant resilience to salt stress, AM effects on these physiological processes are not always predictable. For instance, the symbiosis has resulted in increased proline in host tissues (e.g., Jindal et al., 1993; Zeng et al., 2011; Campanelli et al., 2013), decreased proline (Aboul-Nasr, 1999; He et al., 2010; Evelin et al., 2013) and no effect on proline (Diouf et al., 2005; Borde et al., 2010). The same is true for concentrations of other organic solutes and inorganic cations. Recent reviewers have noted that findings have been inconsistent in regard to mycorrhizal influence on osmoregulation, ion homeostasis and potassium relations (Ruíz-Lozano et al., 2012; Garcia and Zimmermann, 2014).

Many articles offer the conclusion that AM symbiosis altered osmotic or ionic adjustment of the host plant under study. We sought to determine if this is generally true across the published literature and if so, how large the average mycorrhizal effects have been. We conducted a quantitative review of the literature using meta-analysis to synthesize an overall, summary effect size of AM symbiosis on several plant characteristics associated with osmotic adjustment resulting from exposure to NaCl. Meta-analysis is a synthetic approach that enables the reviewer to understand the results of any study in the context of all other related studies. Its purpose is to determine whether the treatment effect of interest is consistent across the body of data, and if it varies substantially from study to study, what might account for this variance (Borenstein et al., 2009). Explanatory variables or “moderators” are often examined in meta-analyses to help understand how experimental circumstances modify the treatment effect of interest. Several researchers have performed meta-analyses on mycorrhizal influences and included numerous moderator variables (e.g., Veresoglou et al., 2012; Mayerhofer et al., 2013; Jayne and Quigley, 2014). We examined nine pre-selected moderators that could potentially influence the size of an AM effect on osmotic solutes. Osmotic adjustment has been viewed in mycorrhizal studies in terms of accumulation of inorganic ions and organic solutes, active production of osmoprotectant molecules, potassium-sodium balance, and changes in host water potential (Ψ) or its components. We focused on these characteristics in the analysis. Our purpose was to answer the following questions:

1. What is the overall AM impact across studies on leaf Ψ, osmotic potential (Ψ_π_) and osmotic solute concentrations on plants exposed to NaCl stress?
2. What is the AM effect on shoot and root K^+^/Na^+^ ratios with exposure to NaCl stress?
3. Have particular symbiont combinations or experimental conditions led to relatively large AM impacts on host osmotic adjustment?

Determining which plant characteristics have been most affected by the symbiosis—and which have been affected most consistently—after exposure to NaCl should help investigators employ AM technology more successfully in cropping and conservation efforts.

## Materials and methods

### Data collection

Using the ISI Web of Science search tool (Thompson Reuters), studies were identified through a systematic search of 11 electronic databases for both refereed and non-refereed articles, including dissertations. On 17 March 2014, we conducted a three-tiered search of these databases through the year 2013. We located 38,318 unique articles with the search terms: “arbuscular mycorrhiza^*^” OR endomycorrhiza^*^ OR “AM mycorrhiza^*^” OR “AM symbios^*^” OR “AM fungi” OR “VAM symbios^*^” OR “VAM fungi” OR “VA mycorrhiza^*^.” A search of these records using the terms salt^*^ OR salin^*^ OR NaCl OR sodium resulted in 948 articles. Of these, 377 articles were obtained using the search terms: proline OR glycine-betaine OR polyamine^*^ OR amino^*^ OR sucrose OR fructose OR glucose OR sugar OR sorbitol OR polyol^*^ OR osmoregula^*^ OR osmotic^*^ OR “water potential” OR potassium OR K+. These 377 articles were extracted from six databases: Web of Science Core Collection, CAB International, MEDLINE, Biological Abstracts, Data Citation Index and Scientific Electronic Library Online (SciELO) databases. With examination of these 377 eligible articles, 270 were excluded because they did not meet our inclusion criteria: AM or NM treatments were not included; salt treatments were not applied to both AM and NM treatments; osmotic adjustment data were not provided (no data on Na+, K+, water potential components or the osmotic solutes of interest, proline, polyamines, soluble sugars, etc); NaCl content of salt treatment was not reported; NaCl accounted for less than half (by molarity) of salt treatment composition; they were reviews; they were not obtainable using interlibrary loan services (2 articles). We identified 107 articles that met our screening criteria (full citation list and details of primary studies provided in Supplementary Material 1). Papers spanned 30 years and were in English, Chinese, Persian and French.

Treatment means and sample sizes were collected for each study. For publications reporting means for more than one NM control treatment in a non-factorial experiment, we used the NM control that most closely approximated AM plants. If sample size was given as a range, we used the smallest value. For studies that did not report sample size, we used *n* = 1 (1 study) unless LSD or standard errors were provided, in which case we used *n* = 2 (17 studies). Including these studies increased the power of the analysis, with their weight limited by a conservative assignment of sample size. If data were provided in graphical form, means were extracted using WebPlotDigitizer (Rogatgi, 2011).

Multiple treatments or host/symbiont combinations from one article were treated as independent studies and represented an individual unit in the meta-analysis. For example, Hartmond et al. (1987) examined the effects of two salt treatments on each of three host species, which resulted in six studies for the meta-analysis from that article. Al-Karaki et al. (2001) reported data for three levels of salt stress in a salt-tolerant and a salt-sensitive cultivar, resulting in six studies. Although designating multiple studies from one publication has the disadvantage of increasing the dependence among studies that for the purposes of meta-analysis are assumed to be independent (Gurevitch and Hedges, 1999), the greater number of studies maximizes the analysis' statistical power (Lajeunesse and Forbes, 2003). This approach has been used commonly in mycorrhizal and plant biology meta-analyses (e.g., Hoeksema et al., 2010; Holmgren et al., 2012; Veresoglou et al., 2012; Mayerhofer et al., 2013; McGrath and Lobell, 2013). We derived 650 studies from the 107 articles. As in prior meta-analyses (e.g., Mayerhofer et al., 2013), we used the final time point in the meta-analysis, for studies that included data for multiple time points.

### Effect size and moderator variables

We conducted several meta-analyses on the osmotic and ionic adjustment measures. Studies were compared via treatment effect size, which was computed as the natural logarithm of the response ratio (ln *R*) of the mycorrhizal to nonmycorrhizal means:

$$\ln R=\ln Y_{\text{AM}}/Y_{\text{NM}}$$

where *Y*_AM_ and *Y*_NM_ are means of AM treatments and NM controls (Rosenberg et al., 2000). These were used to measure the overall effect: the summary or cumulative AM/NM effect size across studies (Borenstein et al., 2009). It is common to use a response ratio in meta-analyses of plant and mycorrhizal behaviors (e.g., Lehmann et al., 2012; Mayerhofer et al., 2013; Jayne and Quigley, 2014), as it gives a standardized expression of treatment-induced change and has direct biological significance. The log transformation is needed to properly balance positive and negative treatment effects across response ratios (to maintain symmetry in the analysis, Borenstein et al., 2009). For our analyses with response ratios, ln values above 0 indicate an AM-induced increase in the parameter of interest, values below 0 indicate an AM-induced decrease in the parameter, and a value of 0 signifies a lack of mycorrhizal effect.

In addition to measures associated with osmotic adjustment, we recorded information from each study on nine moderator variables, characteristics that may modify response to salinity stress and potentially the degree of AM influence on the response. Each moderator had at least two categories (levels) and the data within each of these levels were collected from at least 3 studies from >1 article (moderator levels are detailed in Supplementary Material 1). These moderators were used as explanatory variables in the meta-analyses of summary effects showing significant heterogeneity. Moderators were chosen to determine if an AM effect has been more pronounced under some conditions than others. For summary effects showing heterogeneity (i.e. the effect of AM symbiosis differed among studies even after random variance was accounted for), we wanted to quantify whether some host taxa have been more sensitive to AM colonization than others, whether different AM fungi have differentially influenced host response, and whether experimental situations may have impacted findings.

If a study gave data for more than one level of NaCl stress, we coded a stress severity moderator, scoring the lowest level “low” and the highest level “high,” in an effort to examine in a separate subgroup meta-analysis whether the characteristics of interest were affected by severity of stress. For studies with >2 levels of NaCl stress, e.g., 50, 100, 150, 200, and 250 mM NaCl, the 50 mM treatment was scored as low and the 250 mM treatment as high. When mineral concentrations or dry weights were provided for whole plants and not for shoots, whole plant values were used in the analysis as reasonable proxies of the AM/NM shoot effect size. To be consistent across the analysis, K^+^/Na^+^ ratios were computed from the AM and NM means provided in each primary study, as several papers did not report K^+^/Na^+^ ratio for individual experimental units. Soluble sugars were coded as soluble carbohydrates in our analysis. Total soluble carbohydrates, when measured by the anthrone-sulphoris colorimetric assay, are considered essentially synonymous with total soluble sugars (e.g., Sharifi et al., 2007; Leyva et al., 2008). The host water status moderator was characterized by leaf Ψ and/or relative water content (RWC). A single species was included as a level in the AM taxa moderator if at least 8 studies and >1 paper reported data for it. Those species in <8 studies or just 1 paper were grouped into “other species.”

### Meta-analysis

We estimated the summary effect (mean effect size across studies) with Comprehensive Meta-Analysis (CMA) software (Version 3, Biostat, Englewood, NJ, USA; 2014). Individual studies within the meta-analyses were weighted using non-parametric variance:

$$V_{\text{ln }R}=\left(n_{\text{AM}}+n_{\text{NM}}\right)/\left(n_{\text{AM}}*n_{\text{NM}}\right)$$

where *V*_ln *R*_ is the variance of the natural log of the response ratio *R* and n_AM_ and n_NM_ are the samples sizes of the AM and NM treatments (Rosenberg et al., 2000). Several publications did not report standard errors or standard deviations, nor was sufficient information given in many instances to estimate these from LSD or other mean separation test values. As has often been noted (e.g., Adams et al., 1997; Lehmann et al., 2012; Veresoglou et al., 2012; Mayerhofer et al., 2013), it is not uncommon for measures of dispersion to have been omitted from publications involving plants, which makes calculating weighting based solely on sample size (non-parametric variance) a necessity. Excluding studies that report sample size but not some measure of dispersion would represent a substantial loss of analytical power.

Heterogeneity was assessed with the *Q* statistic (a measure of weighted squared deviations), which shows presence versus absence of heterogeneity, and quantified using *I*^2^, a descriptive index that estimates the ratio of true heterogeneity to total heterogeneity across the observed effect sizes (Higgins and Thompson, 2002; Huedo-Medina et al., 2006). Total heterogeneity (*Q*_*t*_) is composed of expected variation (*Q*_*w*_; within-study heterogeneity, or sampling error) and excess variation (*Q*_*m*_; true heterogeneity in effect sizes among studies) (Borenstein et al., 2009). *I*^2^ is defined as (*Q*_*t*_ − *df*)/*Q*_*t*_, where degrees of freedom (*df*) represents expected variation and *Q*_*t*_ − *df*, true heterogeneity. Negative values of *I*^2^ are set equal to zero so that *I*^2^ lies between 0 and 100%. A value of 0% indicates no true heterogeneity, positive values indicate true heterogeneity in the data set with larger values reflecting a larger proportion of the observed variation due to true heterogeneity among studies. Assumptions of homogeneity were considered invalid when *p* values for the *Q*-test (chi-square test) for heterogeneity were less than 0.1 (e.g., Bristow et al., 2013; Iacovelli et al., 2014). When summary effect sizes displayed true heterogeneity (positive *I*^2^ values; the observed variance could not be explained solely by sampling error), they were examined using the random-effects model, to test for differences in the summary effect among moderator groups, examining *p* values associated with the between-class heterogeneity, *Q*_*m*_. The mean effect size of a moderator or level of a moderator was considered significant if its 95% CI did not overlap zero and if the *p* value was < 0.05. We assumed a common among-study variance across moderator subgroups (Borenstein et al., 2009).

Meta-regression analysis was conducted using a random-effects model in CMA (restricted maximum likelihood, Knapp-Hartung method; IntHout et al., 2014) to test associations between effect sizes showing true heterogeneity and the two quantitative moderators, root colonization (percent) and time of exposure to NaCl (days). Whereas categorical moderators are described by discrete categories or levels, quantitative moderators possess numerical values from each study. Meta-regression produces intercept and slope estimates, where the intercept is the summary effect size when the moderator is zero, and the slope is the change in effect size per one unit increase in the moderator. The meta-regression *p*-value tests if this slope is equal to zero. In this context, *I*^2^ is an estimate of the true heterogeneity explained by the quantitative moderator.

Potential publication bias was assessed statistically with Begg and Mazumdar rank (Kendall) correlation and represented graphically with funnel plots of effect sizes versus their standard errors (Begg and Mazumdar, 1994; Borenstein et al., 2009).

## Results

### Overall summary effects

**Figure 1:**
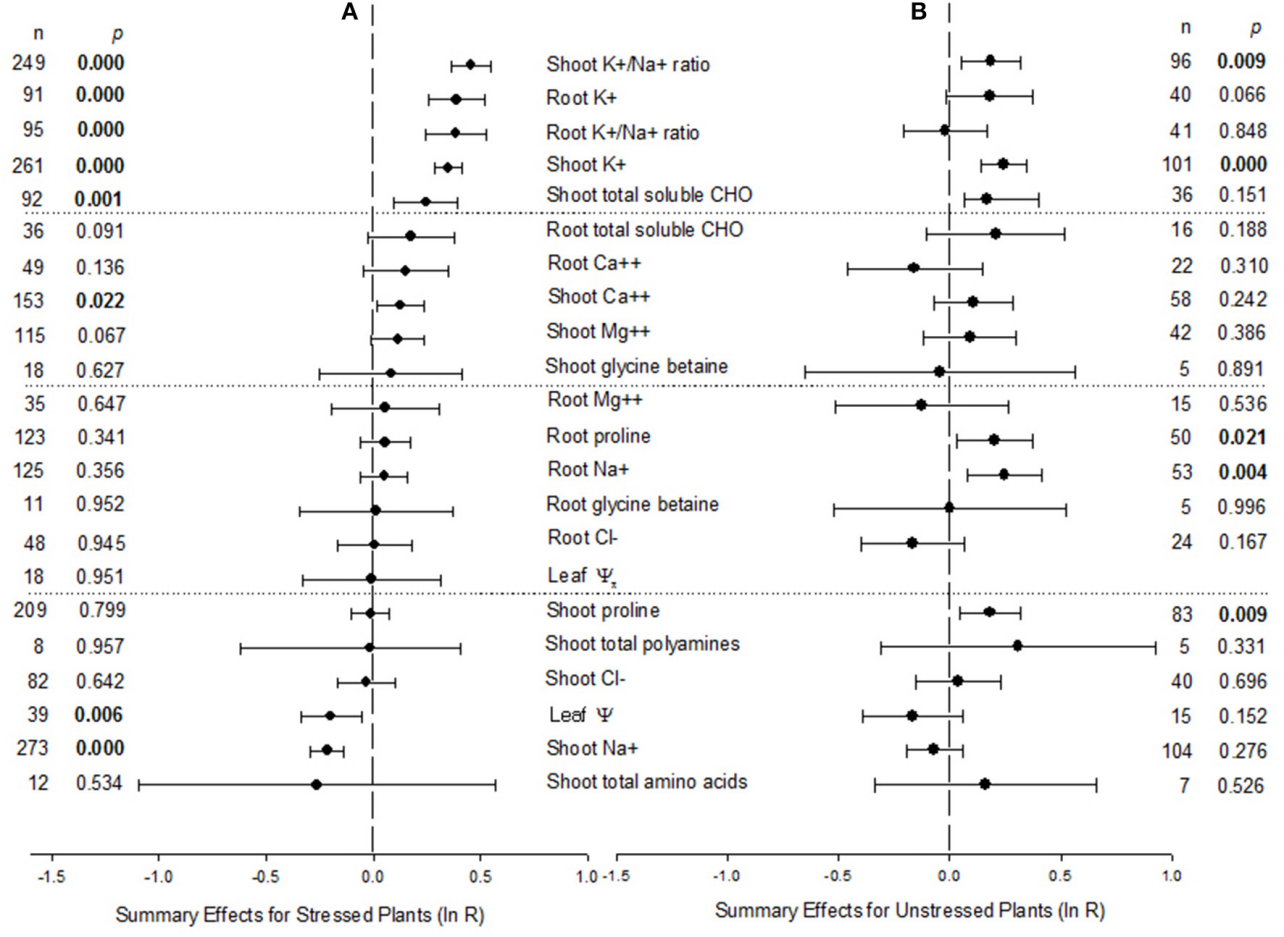
**Weighted summary effect sizes (ln *R*) and 95% bootstrapped confidence intervals (CIs) for AM effect on plants exposed to NaCl stress (A) and unstressed controls (B)**. *p* ≤ 0.05 indicates that the moderator level was significantly different than zero. The absent forest plot for leaf Ψ_π_ in **(B)** reflects insufficient studies to have included that moderator level in the meta-analysis.

We examined AM influence on 22 effect sizes (AM/NM response ratios) in plants exposed to NaCl stress (Figure 1A). Summary effect sizes for unstressed controls from the NaCl studies are provided for context (Figure 1B). Plant hosts were represented by 60 species and 46 genera in 17 families, across the 650 studies (Supplementary Material 1). The best studied woody host genus was *Acacia* (48 studies). Among herbaceous genera, the most information was available for *Lycopersicon esculentum/Solanum lycopersicum* (72 studies). The data set included 21 AM fungal species in nine genera and six families. *R. intraradices* was the most examined AM symbiont (216 studies), followed by *F. mosseae* (188 studies).

We did not see evidence of publication bias in the meta-analysis parameters commonly used to test for it. Visually, the funnel plots for each of the 22 summary effects showed no pattern that would reflect bias toward not reporting small or negative effect sizes. Large or small studies across the range of standard errors had the expected variability around the common effect size. Within the Begg and Mazumdar rank correlation test, 18 of the 22 summary effect sizes had absolute Kendall tau values below 0.20, indicating no publication bias (no tendency for effect sizes to increase as study size decreases). I.e., there was no indication that in order for small studies to be published, they needed to have relatively larger effects sizes. The other four had absolute Kendall tau values above 0.40, but low sample sizes or funnel plots indicated little concern about publication bias.

**Figure 2:**
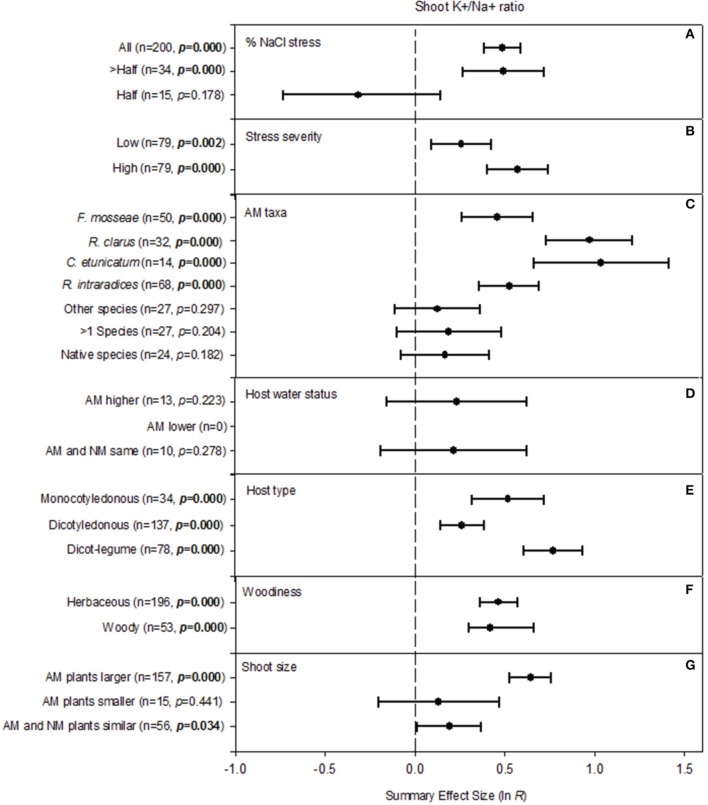
**Weighted summary effect sizes (ln *R*) and 95% bootstrapped confidence intervals (CIs) for influence of NaCl stress on shoot K^+^/Na^+^ ratio**. Comparisons among levels of **(A)** % NaCl stress, **(B)** Stress severity, **(C)** AM taxa, **(D)** Host water status, **(E)** Host type, **(F)** Woodiness, and **(G)** Shoot size. *p* ≤ 0.05 indicates that the moderator level was significantly different than zero. The absent forest plot for “AM lower” in **(D)** reflects insufficient studies to have included that moderator level in the meta-analysis.

**Figure 3:**
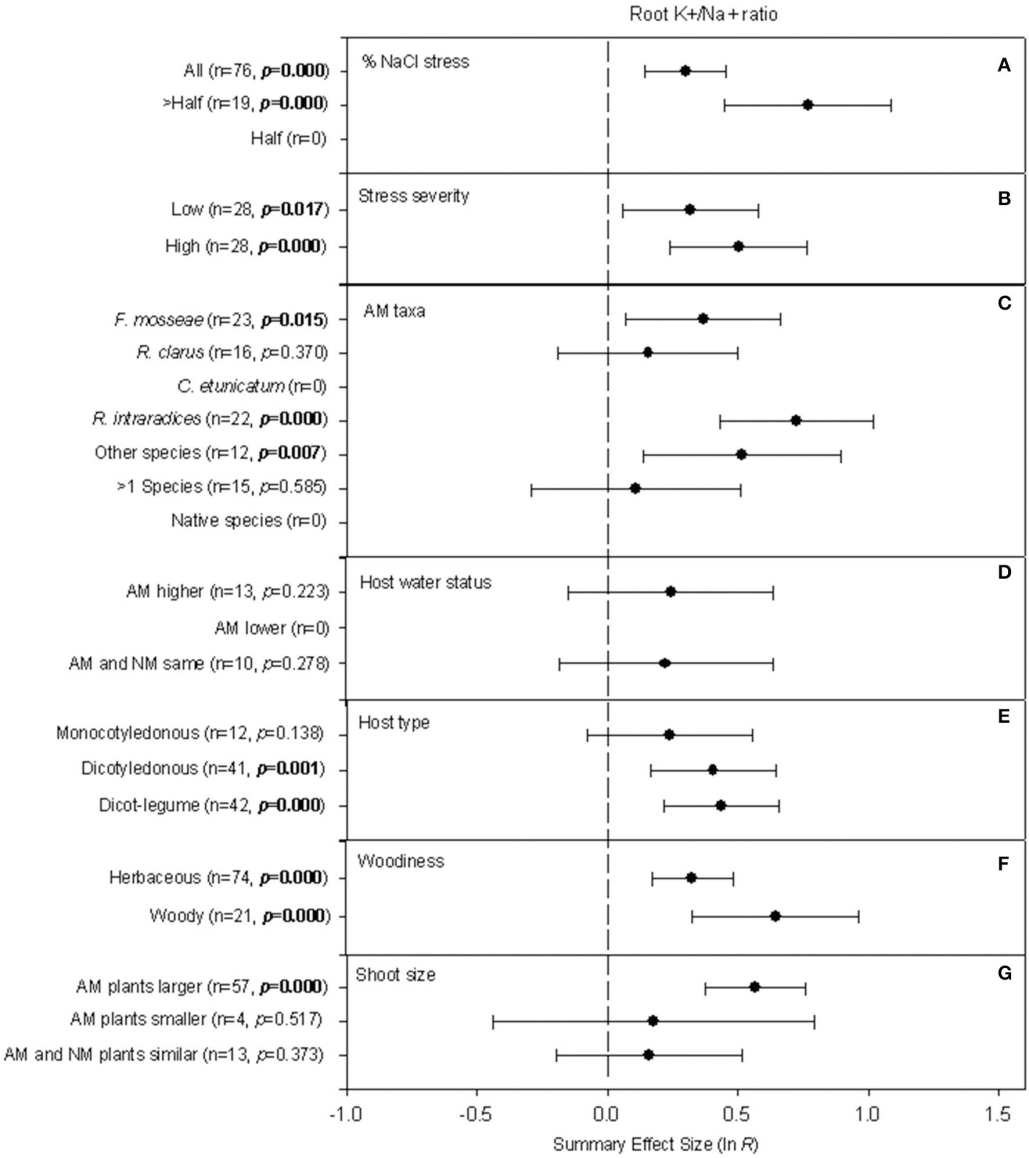
**Weighted summary effect sizes (ln *R*) and 95% bootstrapped confidence intervals (CIs) for influence of NaCl stress on root K^+^/Na^+^ ratio**. Comparisons among levels of **(A)** % NaCl stress, **(B)** Stress severity, **(C)** AM taxa, **(D)** Host water status, **(E)** Host type, **(F)** Woodiness, and **(G)** Shoot size. *p* ≤ 0.05 indicates that the moderator level was significantly different than zero. Absent forest plots for a particular level of a moderator reflects insufficient studies to have included that level in the meta-analysis.

**Figure 4:**
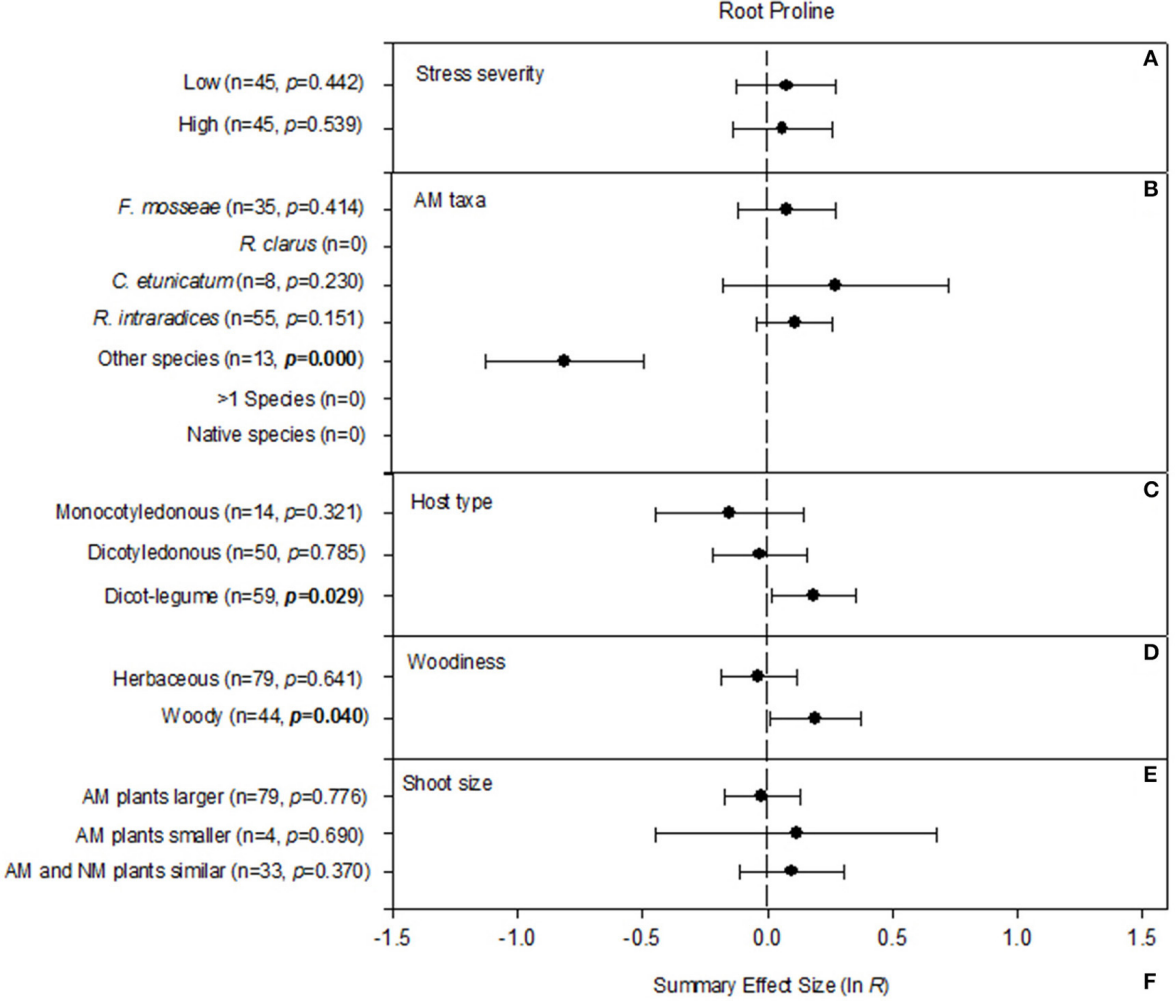
**Weighted summary effect sizes (ln *R*) and 95% bootstrapped confidence intervals (CIs) for influence of NaCl stress on root proline**. Comparisons among levels of **(A)** Stress severity, **(B)** AM taxa, **(C)** Host type, **(D)** Woodiness, and **(E)** Shoot size. *p* ≤ 0.05 indicates that the moderator level was significantly different than zero. Absent forest plots for a particular level of a moderator reflects insufficient studies to have included that level in the meta-analysis.

Natural logs of summary effect sizes are depicted in the forest plots (Figures 1–**4**; Supplementary Material 2), where the zero lines indicate zero mycorrhizal effect. Raw percentage changes induced by AM symbiosis in these summary effects, which can be more intuitive to grasp, are listed in Table 1. AM symbiosis had a significant effect on eight of the 22 host characteristics. The symbiosis significantly stimulated six response ratios and diminished two response ratios. The largest impact was on shoot K^+^/Na^+^ ratio, with a summary effect of 0.457 (raw response ratio = 1.58), a 58% AM-induced increase over the 249 studies that reported these data. Root K^+^/Na^+^ ratio and shoot and root K concentrations were also elevated markedly in AM plants, by 47, 47, and 42%, respectively, relative to their NM counterparts. Total shoot soluble carbohydrate concentrations were on average 28% higher in AM than in NM plants. The 13% increase in Ca^++^ concentration in shoots of AM plants was also significantly different than zero. The symbiosis decreased leaf Ψ and shoot Na^+^ concentration, each by about 18%.

**Table 1:** **Heterogeneity statistics for the 22 summary effect sizes under NaCl stress: *Q*_*t*_, total heterogeneity; *p*_hetero_, probability that the observed heterogeneity was due entirely to sampling error and not to variation among true effects; *I*^2^, percentage of heterogeneity due to true variation among effect sizes**.

**Summary effect**	***Q_t_***	***p*_hetero_**	***I*^2^**	**AM-induced change (%)**
**Shoot K^+^/Na^+^ ratio**	**313**	**0.003**	**21**	**58**
Root K^+^	93	0.399	3	47
**Root K^+^/Na^+^ ratio**	**121**	**0.031**	**22**	**47**
Shoot K^+^	158	1.000	0	42
Shoot total soluble CHO	63	0.988	0	28
Root total soluble CHO	2	1.000	0	19
Root Ca^++^	12	1.000	0	16
Shoot Ca^++^	41	1.000	0	13
Shoot Mg^++^	25	1.000	0	12
Shoot glycine betaine	4	1.000	0	9
Root Mg^++^	2	1.000	0	6
**Root proline**	**151**	**0.037**	**19**	**6**
Root Na^+^	69	1.000	0	5
Root glycine betaine	2	0.996	9	1
Root Cl^−^	8	1.000	0	1
Leaf Ψ_π_	2	1.000	0	−1
Shoot proline	182	0.904	0	−1
Shoot total polyamines	<1	1.000	0	−2
Shoot Cl^−^	30	1.000	0	−3
Leaf Ψ	3	1.000	0	−18
Shoot Na^+^	135	1.000	0	−19
**Shoot total amino acids**	**49**	**<0.001**	**77**	**−23**

*Summary effect sizes showing significant heterogeneity among true effects (p ≤ 0.1) are shown in bold. Summary effects are ordered from high to low, as in Figure 1A. For AM-induced changes in summary effects, computed from ln R as raw percentages, positive values indicate AM-induced increases and negative values AM-induced decreases. Sample sizes and p-values for summary effect sizes differing from zero are given in Figure 1A*.

Across studies, a significant AM impact was not seen on several summary response ratios. This does not mean that the symbiosis did not affect these host characteristics; it means that to date there is insufficient statistical power to demonstrate a significant effect. For the AM-induced increases of 19, 16, and 12%, respectively, on total root soluble carbohydrates, root Ca^++^ concentration and shoot Mg^++^ concentration, CIs included 0, but just marginally. For many of the other host characteristics, the AM effect appeared negligible; ten showed a mycorrhizal impact of less than 10%, with a fairly sizeable overlap of CIs with zero. AM symbiosis reduced total shoot amino acid concentration by 23% on average over the 12 studies reporting these data, but there was large variation. AM symbiosis also had significant impacts on five host characteristics in the absence of salt stress (Figure 1B). Shoot and root proline concentrations, and shoot K^+^/Na^+^ ratio, were each about 20% higher in AM than NM plants. Shoot K^+^ and root Na^+^ concentrations were each 28% higher in AM plants.

### Moderator variables with significant heterogeneity

Heterogeneity *p*_hetero_ values were significant (*p*_hetero_ < 0.100) for four of the 22 summary effect sizes depicted in Figure 1A, and these summary effects also had positive *I*^2^ values: shoot K^+^/Na^+^ ratio (*p*_hetero_ = 0.003, *I*^2^ = 21%), root K^+^/Na^+^ ratio (*p*_hetero_ = 0.031, *I*^2^ = 22%), root proline concentration (*p*_hetero_ = 0.037, *I*^2^ = 19%) and total amino acid concentration of shoots (*p*_hetero_ < 0.001, *I*^2^ = 78%) for NaCl-stressed plants (Table 1). When true effect sizes differ among studies, the source of this real or true heterogeneity is often investigated with moderator or subgroup analysis. For the 18 summary effects not having significant heterogeneity (Figure 1A), *p*_hetero_ was 0.99 or 1.00 for 17 of them, with *I*^2^ = 0.0 for these 17. *I*^2^ had a small positive value (3) for root K concentration, with *p* = 0.399 and hence no significant heterogeneity. None of the 22 summary effects in unstressed plants showed significant heterogeneity. Because the analysis indicated that there was true variation among four effect sizes, results are given for the random-effects model for these summary effects. The fixed-effects model was appropriate for the other 18 summary effects with NaCl stress and all of the summary effects in unstressed conditions (note: meta-analysis summary statistics for the fixed effects model equal those for the random effects model when there is no true between-studies heterogeneity). To investigate sources of this heterogeneity, we conducted moderator analysis of these effect sizes (Table 2; Figures 2–**4**). Moderator analysis was not performed on shoot amino acid concentration due to insufficient studies within moderator levels for this effect size.

**Table 2:** Categorical and regression moderators examined for the three^*^ summary effects showing significant true heterogeneity among effect sizes.

	**Shoot K^+^/Na^+^ ratio**					**Root K^+^/Na^+^ ratio**					**Root proline**				
**Categorical Moderator**	***Qm***	***n***	***p*_hetero_**	***df***	***I*^2^**	***Q_*m*_***	***n***	***p*_hetero_**	***df***	***I^2^***	***Q_*m*_***	***n***	***p*_hetero_**	***df***	***I*^2^**
**A**															
% NaCl in salt stress^***^	11.4	249	0.003	2	82.4	6.7	95	0.009	2	85.1					
Stress severity	6.8	158	0.009	1	85.2	1.0	56	0.326	1	0.0	0.0	90	0.913	1	0.0
AM taxa^**^	43.7	242	<0.001	6	86.3	9.0	88	0.061	4	55.7	28.7	111	<0.001	3	89.5
Host water status^***^	<0.01	23	0.952	1	0.0	0.0	23	0.952	1	0.0					
Host type	23.7	249	<0.001	2	91.6	1.1	95	0.591	2	0.0	4.9	123	0.086	2	59.2
Woodiness	0.2	249	0.697	1	0.0	3.1	95	0.077	1	68.0	3.5	123	0.060	1	71.7
Shoot size	21.8	248	<0.001	2	90.8	4.7	74	0.095	2	57.5	0.9	116	0.636	2	0.0
**Regression Moderator**	***n***	**intercept**	**slope**	***p*_hetero_**	***I*^2^**	***n***	**intercept**	**slope**	***p*_hetero_**	***I*^2^**	***n***	**intercept**	**slope**	***p*_hetero_**	***I*^**2**^**
**B**															
**Time of exposure**	234	0.691	−0.004	0.070	14.0	88	0.964	−0.010	0.002	18.6	123	−0.196	0.005	0.015	15.8
**Root colonization**	225	0.222	0.006	0.009	17.3	85	0.113	0.005	0.172	25.2	118	0.153	−0.002	0.413	16.0

*Q_m_ = between study heterogeneity; n = sample size; p_hetero_ = probability that all observed (total) heterogeneity is due to sampling error (expected variation); df = degrees of freedom, levels within a moderator; I^2^ = percentage of total heterogeneity due to true differences in summary effects among levels of a moderator. The levels of of each categorical moderator with their summary effect sizes, CIs, number of studies and significance values are given in Figures 2–**4***.

* *Total amino acid concentration of shoots also showed significant heterogeneity but there were not enough studies within moderator levels to perform moderator analysis for this summary effect*.

** A single species was included as a level in the AM taxa moderator if at least 8 studies from >1 paper reported data for it. Those species with data from <8 studies or just 1 paper were grouped into “other species.”

*** *% NaCl in salt stress and Host water status are blank for root proline because of insufficient articles providing these data*.

#### Shoot K^+^/Na^+^ ratio

Five of the seven categorical moderators significantly affected how AM symbiosis changed the shoot K^+^/Na^+^ ratio (Table 2; Figure 2). i.e., different levels within each of these five moderators led to different AM impacts on shoot K^+^/Na^+^. When NaCl was responsible for over half or all of the salt stress treatment, AM symbiosis increased shoot K^+^/Na^+^ dramatically, by 64%. When NaCl accounted for half of the stress, the summary effect was −27% (not significant). Stress severity also explained a significant amount of heterogeneity (Figure 2B). The AM influence was much more pronounced at relatively high NaCl concentrations (78% AM-induced increase) compared to the low NaCl concentration treatments (30% AM-induced increase). Which AM taxa colonized roots had an appreciable influence on the size of the AM effect (Figure 2C). Plants colonized by *C. etunicatum* and *R. clarus* had 182 and 164% larger shoot K^+^/Na^+^, respectively, than uncolonized plants. *R. intraradices* and *F. mosseae* symbioses resulted in 69 and 58% higher shoot K^+^/Na^+^. Colonization by other single species, colonization by >1 species and colonization by unidentified native fungi each increased the summary shoot K^+^/Na^+^ ratio, but the increase was not significant. Host water status (characterized as leaf Ψ and/or RWC) was not reported in many studies. Where it was reported, whether it was higher or lower in AM plants relative to NM plants did not change the AM effect on shoot K^+^/Na^+^ (Figure 2D). Across studies, host type had a substantial influence on the mycorrhizal effect on shoot K^+^/Na^+^ (Figure 2E). The AM effect was about 4× larger in legumes (116%) than in non-legume dicots (30%). Monocotyledonous hosts averaged 68% higher shoot K^+^/Na^+^ when mycorrhizal. The AM effect was similar in woody and herbaceous plants (Figure 2F). AM effect on plant size was associated with AM effect on shoot K^+^/Na^+^ ratio (Figure 2G). When AM plants were larger than NM plants, the AM effect on shoot K^+^/Na^+^ was much larger (90% increase) than when AM and NM plants were similar in size (21%; significant effect) or when AM plants were smaller (14%; insignificant effect).

#### Root K^+^/Na^+^ ratio

Four of the seven categorical moderators significantly affected how AM symbiosis changed the K^+^/Na^+^ ratio in roots (Table 2; Figure 3). Three of these are the same moderators that significantly moderated AM impact on shoot K^+^/Na^+^. When NaCl was responsible for the majority of the salt treatment (over half but not all of the treatment), AM symbiosis increased root K^+^/Na^+^ by 115% (Figure 3A). In experiments in which NaCl comprised all of the salinity stress, the summary effect was still substantial but much smaller, at 35%. AM-induced increases in root K^+^/Na^+^ ratio ranged from 35 to 66% based on stress severity (Figure 3B) but the difference was not significant (Table 2). AM-induced increases in root K^+^/Na^+^ ratio ranged from 12 to 107% based on AM taxa (Figure 3C), and this heterogeneity was significant (Table 2). *R. intraradices* had the largest effect on root K^+^/Na^+^ (average increase of 107%), followed by *F. mosseae* (45%) and *R. clarus* (17%). Less studied fungal species (grouped as “other species” for the meta-analysis) had an average summary effect of 68%, and experiments with hosts colonized by >1 species, 12%. Larger root K^+^/Na^+^ ratios in AM plants were not affected by host water status, with summary effects about 25% whether water status was higher in AM plants or the same in AM and NM plants (Figure 3D). The AM effect was about twice as high in dicots (50–55%) as in monocots (27%) (Figure 3E), though the *p*-value of 0.59 did not indicate that these summary effects were significantly different. The AM-induced stimulation of root K^+^/Na^+^ ratio was much more pronounced across woody host species (91%) than across herbaceous species (39%) (Figure 3F). As for shoots, K^+^/Na^+^ in roots was affected by mycorrhizae much more when AM plants were larger than their experimental NM controls (Figure 3G).

#### Root proline concentration

For two of the categorical moderators, percent NaCl in the stress treatment and host water status, there were insufficient studies for two of the three levels of the moderator and so they were not included in the meta-analysis (Table 2, Figure 4). Three of five categorical moderators significantly affected how AM symbiosis changed proline concentrations in roots. The insignificant AM-induced increase in root proline was similar among the three most-studied single AM species, while other AM species substantially reduced root proline concentration (Figure 4B). There was also significant heterogeneity in the summary effects of the host type and woodiness moderators. Monocotyledonous hosts have shown on average 14% reduction in root proline concentration when colonized by AM fungi, non-legume dicotyledonous hosts virtually no AM effect and legumes a 20% increase when mycorrhizal (Figure 4C). Herbaceous hosts have shown negligible response to the symbiosis and woody hosts an overall 20% increase in root proline when mycorrhizal (Figure 4D). Stress severity (Figure 4A) and shoot size (Figure 4E) did not change the effect of AM symbiosis on root proline.

Differing amounts of K fertilization to AM and NM plants was originally included as one of our pre-specified moderator variables, but we located only two articles containing data for the above three effect sizes (those showing significant heterogeneity) that reported giving differential K fertilization to experimental plants: Graham and Syvertsen (1989) for shoot K^+^/Na^+^ ratio and Ruíz-Lozano et al. (1996) for root proline concentration. This did not meet the coding criteria and K fertilization was excluded as a moderator. However, as giving one treatment more K than another would be expected to alter the K^+^/Na^+^ ratio, regardless of AM symbiosis, we felt it was important to determine if these studies may have affected these effects. We conducted meta-analyses with and without the data from these articles (sensitivity analysis, Higgins and Green, 2011). Despite NM plants receiving more K in these experiments, removing the data resulted in a slightly higher summary AM-induced shoot K^+^/Na^+^ ratio (63% increase without the data from the one article vs. 58% with the data) as well as a slightly higher summary AM-induced proline concentration (8% increase without the data from the one article vs. 6% with the data). Significance denoted by *p*-values was not changed in either case.

### Meta-regression

**Figure 5:**
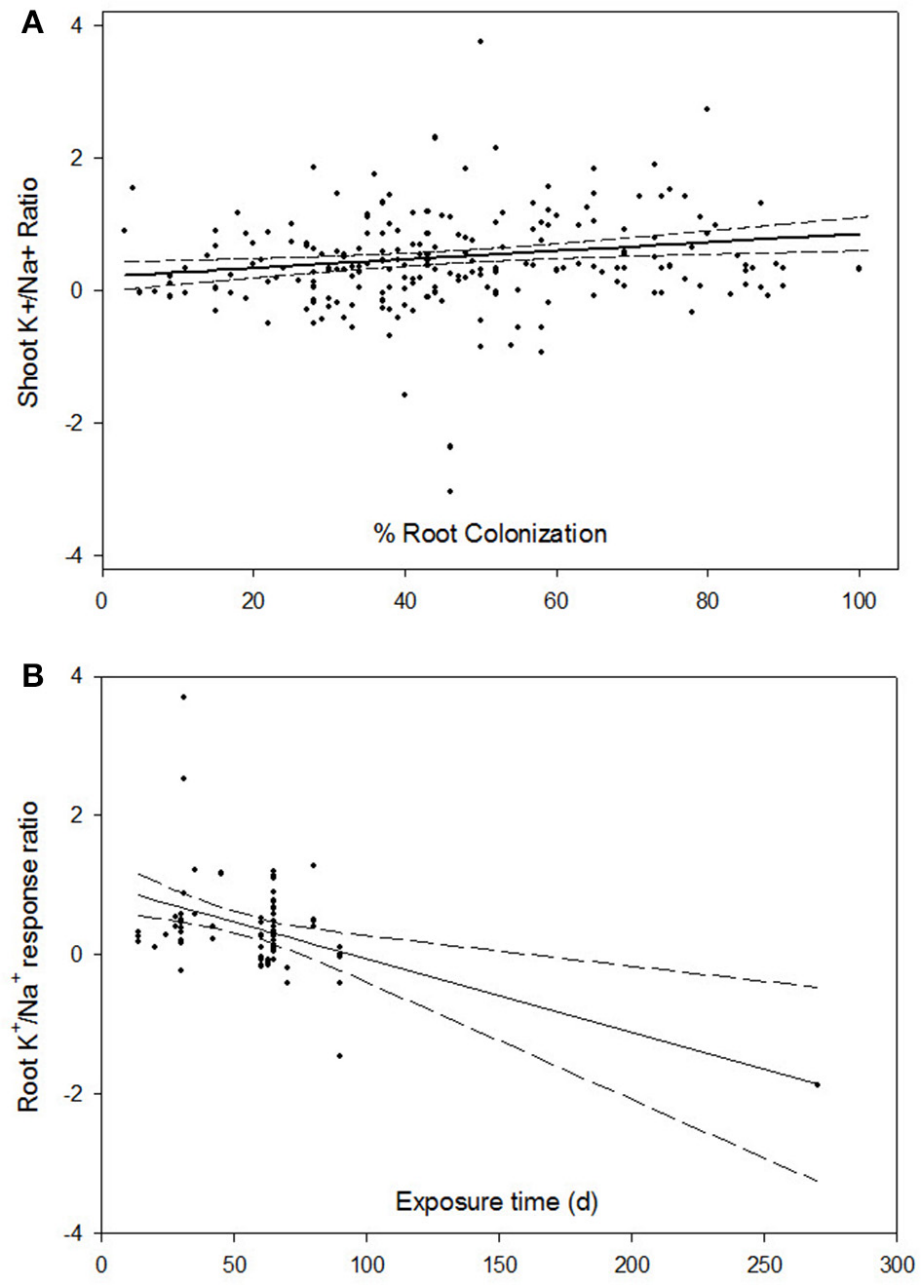
**Natural log of the response ratio of shoot K^+^/Na^+^ as percent root colonization of plants increases (A) and root K^+^/Na^+^ as time of exposure to NaCl increases (B)**. Dotted lines above and below the predicted slope are the upper and lower 95% confidence intervals.

In addition to the seven categorical moderators summarized above, we examined two regression moderators. For these, rather than assigning a category or level to the moderator, values for degree of root colonization and time of exposure to the NaCl stress for each primary study were included in meta-regression analysis. Meta-regression of shoot K^+^/Na^+^ ratio showed a positive response to percent root colonization, with ln *R* increasing by 0.006 (*p* = 0.009) for each percent increase in root colonization. Figure 5A illustrates this relationship, and the consistent response across studies is apparent. Natural log of the response ratio was predicted to increase from 0.222 to 0.822 as colonization increases from 0 to 100%. Time of exposure had a substantially negative relationship on root K^+^/Na^+^ ratio, dropping from +1 for short exposure studies to −1 after 80 days of exposure (Figure 5B). However, due to high variability among studies with similar exposure times, the slope was statistically zero (*p* = 0.070). Interestingly, if the meta-regression was conducted without the extreme 270 d study (Scheloske et al., 2004), this study's effect size was predictable from the other studies. Root proline concentration had the opposite response to time of exposure, with ln *R* increasing by 0.005 (*p* = 0.015) per day. The response of root K^+^/Na^+^ ratio and root proline to percent root colonization was essentially zero (*p* = 0.172 and *p* = 0.413, respectively). Root colonization and time of exposure were not significantly correlated; *r*^2^ = 0.001 for 407 study data pairs.

## Discussion

AM symbiosis often increases plant resilience to environmental stresses, including temperature extremes (Zhu et al., 2010; Maya and Matsubara, 2013), phytoxicity (Seguel et al., 2013), heavy metals (Forgy, 2012), soil compaction (Miransari, 2013), drought (Augé, 2001; Querejeta et al., 2006), disease (Nadeem et al., 2014) and insect herbivory (Jung et al., 2012). Hundreds of studies have implicated several AM species in promoting resistance to salinity (Ruíz-Lozano et al., 2012; Estrada et al., 2013; Hajiboland, 2013). Modes of AM influence include better mineral nutrition, enhanced osmotic adjustment and ionic adjustment, increased antioxidant production, and greater production and deployment of protective molecules (e.g., membrane and protein protection) (Aggarwal et al., 2012; Ruíz-Lozano et al., 2012). We focused on osmoregulation in this meta-analysis, examining inorganic and organic osmolytes considered to be important in tissue water relations and ionic homeostasis.

### Water relations

Authors of mycorrhizal articles have used the terms osmotic adjustment and osmoregulation to refer both to increased concentrations of specific ions or compatible organic solutes as well as to the overall changes in tissue Ψ components brought about by active accumulation of solutes. There are relatively few data in the published literature for leaf or root Ψ, Ψ_π_ or turgor potential (Ψ_p_) of AM and NM plants subjected to NaCl stress: only seven articles have reported Ψ data and four articles Ψ_π_ data (Supplementary Material 1). Many more articles provide data for individual solutes or groups of solutes such as proline, soluble carbohydrates and inorganic cations. Despite claims that AM symbiosis stimulates osmotic adjustment, the meta-analysis reveals no overall AM effect on leaf Ψ_π_, the Ψ component most often associated by water relations researchers with osmotic adjustment (e.g., Jones and Gorham, 1983; Morgan, 1984). Active osmotic adjustment allows leaves and roots to cope with saline or dry soils by sustaining water movement into plants, maintaining more normal turgor and overall Ψ. Yet AM symbiosis was actually associated with significantly lower leaf Ψ (an average of 18% lower across studies). It would be useful if future studies of AM symbiosis and osmotic adjustment included measures of tissue water relations.

### Mineral cations

Exposure to NaCl injures plants in part through lowered soil Ψ and the ensuing osmotic stress, but ultimately it may be more injurious via direct toxicity of Na^+^ ions. Na^+^ competes with K^+^ for binding sites and thereby interferes with a number of key physiological functions that rely on K^+^ (Evelin et al., 2009), such as stomatal operation, transcription and enzyme function (Blaha et al., 2000; Munns et al., 2006). How effectively a plant can control uptake, distribution and compartmentalization of Na^+^ is a determinant of its salt resistance (Yokoi et al., 2002). The importance of maintaining suitable K^+^/Na^+^ ratios for proper metabolic functioning has become widely accepted as a gauge of NaCl sensitivity in recent years (Ruíz-Lozano et al., 2012). Most mycorrhizal salinity stress papers have included data on tissue K^+^ and Na^+^ concentrations, and the meta-analysis shows that AM symbiosis has had the greatest impact on leaf and root K^+^/Na^+^ ratio and K^+^ concentrations among the 22 effect sizes we examined. Under NaCl stress, AM symbiosis can increase K^+^ absorption and reduce translocation of Na^+^ to shoots (Giri et al., 2007; Sharifi et al., 2007). Weighted across all studies, AM-induced changes have been more pronounced for K^+^ than for Na^+^; the symbiosis has increased K^+^ concentrations in shoots and roots to a greater extent than it has reduced Na^+^ concentrations in these organs. In a research field where experimental findings can differ depending on host/fungal symbiont combination and several experimental conditions, it is remarkable just how consistently and dramatically the symbiosis has promoted higher K^+^/Na^+^ ratios, with an average 58% increase over the 249 studies reporting shoot K^+^/Na^+^ ratio and 47% increase over the 95 studies reporting root K^+^/Na^+^ ratio. Recent reviews have described mechanisms by which AM symbiosis may enable this (Evelin et al., 2009; Aggarwal et al., 2012; Ruíz-Lozano et al., 2012; Hajiboland, 2013). In addition to balancing Na^+^, AM-induced increases in K^+^ may also contribute to greater root hydraulic conductivity (El-Mesbahi et al., 2012) and hence improved water status with osmotic stress.

Increased Na^+^ in the root zone can inhibit uptake of important nutrient cations other than K^+^, and AM symbiosis can ameliorate some of the associated detrimental effects (Ruíz-Lozano et al., 2012). AM fungi have been shown to selectively absorb K^+^ and Ca^++^ and avoid Na^+^, help plants avoid excess Na^+^ uptake and promote higher plant Ca^++^/Na^+^ ratios (Evelin et al., 2009, 2012; Hammer et al., 2011). The collective increase in Ca^++^ concentration associated with AM symbiosis across studies was 16% in roots (49 studies) and 13% in shoots (153 studies). These may constitute meaningful increases for plants in danger of Ca^++^ limitation.

### Compatible organic solutes

Plants accumulate large amounts of different compatible solutes in response to environmental stresses (Serraj and Sinclair, 2002). Compatible solutes are low molecular weight, highly soluble organic compounds that are typically non-injurious at high cellular concentrations (Hayat et al., 2012). They contribute to osmotic adjustment, detoxification of damaging reactive oxygen species, protection of membrane integrity and protein stabilization (Yancey, 1994). For various plants these can include proline and other amino acids, sugars, polyols and quaternary ammonium compounds such as glycine betaine and proline betaine (Ashraf and Harris, 2004). Proline is among the most studied and abundant compatible solutes in plant abiotic stress research and has been the most frequently examined organic osmolyte in mycorrhizal NaCl studies.

Both increases and decreases in tissue proline in the face of stress have been ascribed to AM symbiosis. There has been a tendency to interpret each as a benefit of the symbiosis: higher levels in AM plants as bolstering a protective compound, and lower levels in AM plants as an indication of more injury to NM plants. Proline plays several major roles during salt stress (Chen and Dickman, 2005; Hayat et al., 2012; Ruíz-Lozano et al., 2012). It acts as a metal chelator, a signaling molecule, and an antioxidative defense molecule that helps buffer cellular redox potential and scavenge free radicals. It is an osmotically active solute that aids in maintaining cell turgor or osmotic balance. It acts to stabilize subcellular structures. Increases in tissue proline have also been viewed as an incidental consequence of NaCl exposure or as a symptom of greater injury in less tolerant plants (Moftah and Michel, 1987; Ruíz-Lozano et al., 2012). Ruíz-Lozano et al. (2012) illustrated the complexity of plant response to AM symbiosis by noting a study with droughted lettuce, in which NM plants accumulated more proline in their shoots than AM plants (Ruíz-Lozano et al., 2011). In contrast, AM lettuce plants accumulated more proline in the roots than NM plants. The interpretation was that, in root tissues, AM plants accumulated more proline to better cope with the low Ψ of dry soil and to maintain a Ψ gradient favorable to water absorption by the roots (e.g., Porcel and Ruíz-Lozano, 2004). Across the 209 studies and 123 studies in our meta-analysis of proline in shoot and roots, respectively, the summary effects indicate no significant association between AM symbiosis and proline concentrations for either organ. The strength of interpretations about AM-induced benefits regarding proline is evidently best gauged within the context of the individual studies.

Glycine betaine can protect plants subjected to saline stress by contributing to osmotic regulation and by protecting the photosynthetic apparatus (Jagendorf and Takabe, 2001). Across the few studies providing these data, glycine betaine concentrations have not been elevated much (and not significantly) by AM symbiosis in plants exposed to NaCl stress. Only one paper that fit our search criteria contained proline betaine data, and so this compatible solute was not a part of the meta-analysis. Soluble carbohydrates/sugars, another class of important osmotic regulators, have been elevated in AM plants to a much greater degree than betaines, second only to K^+^ concentration in terms of size of AM effect across studies. Polyamines may play a role in plant and fungal resistance to a variety of environmental stresses including salinity (Rai and Takabe, 2006; Groppa and Benavides, 2008; Valdés-Santiago and Ruiz-Herrera, 2014), and it has been suggested that modulating polyamine pools in plant tissues may be one way that AM symbiosis increases plant ability to grow in saline conditions (Sannazzaro et al., 2007). In some instances total shoot polyamine concentrations have been higher in AM plants than NM controls (Sannazzaro et al., 2007; Evelin et al., 2013), but over the comparatively few studies containing data for this osmolyte, on average AM and NM plants have not differed after NaCl stress.

### Symbiont taxa

The analysis showed that across all studies, different AM taxa have had significantly different effects on shoot and root K^+^/Na^+^ and on root proline concentration. This has also been demonstrated within individual studies that compared AM symbionts (e.g., Ruiz-Lozano and Azcón, 2000). Symbiont efficiencies have differed with plant-fungus combinations in drought work, as well (Ruiz-Lozano et al., 1995). The fungi themselves have demonstrated large variations in salt tolerance among species and isolates (Rosendahl and Rosendahl, 1991). In attempting to explain why the size of the AM effect on shoot K^+^/Na^+^ ratio differs in different salt stress studies, the meta-analysis shows that the fungi have been more effective in elevating K^+^ and/or excluding Na^+^ not just when overall Na^+^ concentration is especially high, but also when the proportion of Na^+^ ions in the stress treatment is relatively high. This was revealed by the moderator analysis as higher summary AM/NM effect sizes when Na^+^ accounted for more than half of the salt stress (vs. half) and for high severity (vs. low). AM-induced increases in shoot K^+^/Na^+^ ratio were higher in legumes than in monocotyledonous plants or non-legume dicots. Potassium needs tend to be high for legumes (e.g., Kafkafi et al., 1977; James et al., 1995), so this may represent a particular advantage of mycorrhizae for legumes.

### Root colonization

In osmotic stress studies and other physiological areas of mycorrhizal investigation, AM effects have often not been well-correlated with degree of root colonization (e.g., Dakessian et al., 1986; Fitter and Merryweather, 1992; Ruiz-Lozano et al., 1995). We explored this relationship for shoot K^+^/Na^+^ ratio, the summary effect most affected by AM symbiosis among the 22 examined, and did find a significant association with root colonization. The AM effect is substantial; for example, a 51% increase in shoot K^+^/Na^+^ from 20 to 80% colonization. Given the large number of host and fungal species involved, as well as the diversity of experimental conditions (climate, soils, severity of NaCl treatment, time of exposure, fertilization schemes, etc.), it is noteworthy that a significant positive relationship was found across the 225 studies providing these data.

## Conclusions

Salt stress inhibits plant growth in two phases: water deficit caused by lowered Ψ of the soil solution, followed by excessive salts moving in the transpiration stream to leaves which causes injury there (Munns et al., 2006). AM symbiosis has been shown to consistently assist hosts in dealing with each phase. The symbiosis is advantageous to host plants subjected to the osmotic stress of drought and saline conditions (Ruíz-Lozano and Aroca, 2010b; Augé et al., 2014), and it consistently increases shoot K^+^ and decreases shoot Na^+^, as demonstrated by several investigators (Supplementary Material 1) and the outcome of this meta-analysis. AM-induced promotion of salt tolerance has broad ecological and agricultural implications. In dryland agriculture, increased salt tolerance can result in higher yields in saline soils (Munns et al., 2006). In irrigated agriculture, better salt tolerance can decrease the leaching requirement and hence reduce costs (Pitman and Läuchli, 2002). Continued interest in AM symbiosis as an aid to plants faced with salt stress appears well-justified.

### Conflict of interest statement

The authors declare that the research was conducted in the absence of any commercial or financial relationships that could be construed as a potential conflict of interest.

## References

[B1] Aboul-Nasr A. (1999). Alleviation of salt stress by *Glomus intraradices* on linseed (*Linum usitatissimum* L.) in hydroponic culture. Alex. J. Agric. Res. 44, 115–127

[B2] Adams D. C. Gurevitch J. Rosenberg M. S. (1997). Resampling tests for meta-analysis of ecological data. Ecology 75, 1277–1283. 10.1890/0012-9658(1997)078[1277:RTFMAO]2.0.CO;2

[B3] Aggarwal A. Kadian N. Neetu K. Tanwar A. Gupta K. K. (2012). Arbuscular mycorrhizal symbiosis and alleviation of salinity stress. J. Appl. Nat. Sci. 4, 144–155

[B4] Al-Karaki G. N. Hammad R. Rusan M. (2001). Response of two tomato cultivars differing in salt tolerance to inoculation with mycorrhizal fungi under salt stress. Mycorrhiza 11, 43–47. 10.1007/s005720100098

[B5] Aroca R. Porcel R. Ruiz-Lozano J. M. (2012). Regulation of root water uptake under abiotic stress conditions. J. Exp. Bot. 63, 43–57. 10.1093/jxb/err266 21914658

[B6] Ashraf M. Harris P. J. C. (2004). Potential biochemical indicators of salinity tolerance in plants. Plant Sci. 166, 3–16. 10.1016/j.plantsci.2003.10.024

[B7] Augé R. M. (2001). Water relations, drought and VA mycorrhizal symbiosis. Mycorrhiza 11, 3–42. 10.1007/s005720100097

[B8] Augé R. M. Saxton A. M. Toler H. D. (2014). Arbuscular mycorrhizal symbiosis alters stomatal conductance of host plants more under drought than under amply watered conditions: a meta-analysis. Mycorrhiza. [Epub ahead of print]. 10.1007/s00572-014-0585-4 24831020

[B9] Begg C. B. Mazumdar M. (1994). Operating characteristics of a rank correlation test for publication bias. Biometrics 50, 1088–1101. 10.2307/2533446 7786990

[B10] Blaha G. Stelzl U. Spahn C. M. T. Agrawal R. K. Frank J. Nierhaus K. H. (2000). Preparation of functional ribosomal complexes and effect of buffer conditions on tRNA positions observed by cryoelectron microscopy. Meth. Enzymol. 317, 292–309. 10.1016/S0076-6879(00)17021-1 10829287

[B11] Borde M. Dudhane M. Jite P. K. (2010). AM fungi influences the photosynthetic activity, growth and antioxidant enzymes in *Allium sativum* L. under salinity condition. Notulae Sci. Biol. 2, 64–71

[B12] Borenstein M. Hedges L. Higgins J. Rothstein J. (2009). Introduction to Meta-Analysis. Wiley: West Sussex

[B13] Boyer J. S. (1982). Plant productivity and environment. Science 218, 443–448. 10.1126/science.218.4571.443 17808529

[B14] Bristow S. M. Bolland M. J. MacLennan G. S. Avenell A. Grey A. Gamble G. D.. (2013). Calcium supplements and cancer risk: a meta-analysis of randomized controlled trials. Br. J. Nutr. 110, 1384–1393. 10.1017/S0007114513001050 23601861

[B15] Campanelli A. Ruta C. DeMastro G. Morone-Fortunato I. (2013). The role of arbuscular mycorrhizal fungi in alleviating salt stress in *Medicago sativa* L. car. Icon. Symbiosis 59, 65–76. 10.1007/s13199-012-0191-1

[B16] Chen C. Dickman M. B. (2005). Proline suppresses apoptosis in the fungal pathogen *Colletotrichum trifolii*. Proc. Natl. Acad. Sci. U.S.A. 102, 3459–3464. 10.1073/pnas.0407960102 15699356 PMC552905

[B17] Dakessian S. Brown M. S. Bethlenfalvay G. J. (1986). Relationship of mycorrhizal growth enhancement and plant growth with soil water and texture. Plant Soil 94, 439–443. 10.1007/BF02374337

[B18] Diouf D. Duponnois R. Ba A. T. Neyra M. Lesueur D. (2005). Symbiosis of *Acacia auriculiformis* and *Acacia mangium* with mycorrhizal fungi and *Bradyrhizobium* spp. improves salt tolerance in greenhouse conditions. Funct. Plant Biol. 32, 1143–1152. 10.1071/FP04069 32689208

[B19] El-Mesbahi M. N. Azcon R. Ruíz-Lozano J. M. Aroca R. (2012). Plant potassium content modifies the effects of arbuscular mycorrhizal symbiosis on root hydraulic properties in maize plants. Mycorrhiza 22, 555–564. 10.1007/s00572-012-0433-3 22370879

[B20] Estrada B. Aroca R. Barea J. M. Ruíz-Lozano J. M. (2013). Native arbuscular mycorrhizal fungi isolated from a saline habitat improved maize antioxidant systems and plant tolerance to salinity. Plant Sci. 201, 42–51. 10.1016/j.plantsci.2012.11.009 23352401

[B21] Evelin H. Giri B. Kapoor R. (2012). Contribution of *Glomus intraradices* inoculation to nutrient acquisition and mitigation of ionic imbalance in NaCl-stressed *Trigonella foenum-graecum*. Mycorrhiza 22, 203–217. 10.1007/s00572-011-0392-0 21695577

[B22] Evelin H. Giri B. Kapoor R. (2013). Ultrastructural evidence for AMF mediated salt stress mitigation in *Trigonella foenum-graecum*. Mycorrhiza 23, 71–86. 10.1007/s00572-012-0449-8 22733451

[B23] Evelin H. Kapoor R. Giri B. (2009). Arbuscular mycorrhizal fungi in alleviation of salt stress: A review. Ann. Bot. 104, 1263–1280. 10.1093/aob/mcp251 19815570 PMC2778396

[B24] Fitter A. H. Merryweather R. W. (1992). Why are some plants more mycorrhizal than others? an ecological inquiry, in Mycorrhizas in Ecosystems, eds Read D. J. Lewis D. H. Fitter A. H. Alexander I. (Wallingord: CAB International), 26–36

[B25] Forgy D. (2012). Arbuscular mycorrhizal fungi can benefit heavy metal tolerance and phytoremediation. J. Nat. Res. Life Sci. Ed. 41, 23–26. 10.4195/jnrlse.2012.0001se20013261

[B26] Garcia K. Zimmermann S. D. (2014). The role of mycorrhizal associations in plant potassium nutrition. Front. Plant Sci. 5:337. 10.3389/fpls.2014.00337 25101097 PMC4101882

[B27] Giri B. Kapoor R. Mukerji K. G. (2007). Improved tolerance of *Acacia nilotica* to salt stress by arbuscular mycorrhiza, *Glomus fasciculatum* may be partly related to elevated K/Na ratios in root and shoot tissues. Microb. Ecol. 54, 753–760. 10.1007/s00248-007-9239-9 17372663

[B28] Graham J. H. Syvertsen J. P. (1989). Vesicular-arbuscular mycorrhizas increase chloride concentration in citrus seedlings. New Phytol. 113, 29–36. 10.1111/j.1469-8137.1989.tb02392.x

[B29] Groppa M. D. Benavides M. P. (2008). Polyamines and abiotic stress: recent advances. Amino Acids 34, 35–45. 10.1007/s00726-007-0501-8 17356805

[B30] Gurevitch J. Hedges L. V. (1999). Statistical issues in ecological meta-analyses. Ecology 80, 1142–1149. 10.1890/0012-9658(1999)080[1142:SIIEMA]2.0.CO;211351001

[B31] Hajiboland R. (2013). Role of Arbuscular mycorrhiza in amelioration of salinity, in Salt Stress in Plants: Signalling, Omics and Adaptations, eds Ahmad P. Azooz M. A. Prasad M. N. V. (New York, NY: Springer Science+Business Media), 301–354

[B32] Hammer E. C. Nasr H. Pallon J. Olsson P. A. Wallander H. (2011). Elemental composition of arbuscular mycorrhizal fungi at high salinity. Mycorrhiza 21, 117–129. 10.1007/s00572-010-0316-4 20499112

[B33] Hartmond U. Schaesberg N. V. Graham J. H. Syvertsen J. P. (1987). Salinity and flooding stress effects on mycorrhizal and non-mycorrhizal citrus rootstock seedlings. Plant Soil 104, 37–43. 10.1007/BF02370622

[B34] Hayat S. Hayat Q. Alyemeni M. N. Wani A. S. Pichtel J. Ahmad A. (2012). Role of proline under changing environments. A review. Plant Signal. Behav. 7, 1–11. 10.4161/psb.21949 22951402 PMC3548871

[B35] He Z. Q. Tang H. R. Li H. X. He C. X. Zhang Z. B. Wang H. S. (2010). Arbuscular mycorrhizal alleviated ion toxicity, oxidative damage and enhanced osmotic adjustment in tomato subjected to NaCl stress. Am. Eur. J. Agric. Environ. Sci. 7, 676–683

[B36] Higgins J. P. T. Green S. (2011). Cochrane Handbook for Systematic Reviews of Interventions 5.1.0. The Cochrane Collaboration. Available online at: cochrane-handbook.org

[B37] Higgins J. P. T. Thompson S. G. (2002). Quantifying heterogeneity in a meta-analysis. Stat. Med. 21, 1539–1558. 10.1002/sim.1186 12111919

[B38] Hoeksema J. D. Chaudhary V. B. Gehring C. A. Johnson N. C. Karst J. Koide R. T.. (2010). A meta-analysis of context-dependency in plant response to inoculation with mycorrhizal fungi. Ecol. Lett. 13, 394–407. 10.1111/j.1461-0248.2009.01430.x 20100237

[B39] Holmgren M. Gómez-Aparicio L. Quero J. L. Valladares F. (2012). Non-linear effects of drought under shade -reconciling physiological and ecological models in plant communities. Oecologia 169, 293–305. 10.1007/s00442-011-2196-5 22083284 PMC3353118

[B40] Huedo-Medina T. B. Sánchez-Meca J. Marín-Martínez F. Botella J. (2006). Assessing heterogeneity in meta-analysis: Q statistic or I^2^ index? Psychol. Methods 11, 193–206. 10.1037/1082-989X.11.2.193 16784338

[B41] Iacovelli R. Alesini D. Antonella Palazzo A. Trenta P. Santoni M. De Marchis L.. (2014). Targeted therapies and complete responses in first line treatment of metastatic renal cell carcinoma. A meta-analysis of published trials. Cancer Treat. Rev. 40, 271–275. 10.1016/j.ctrv.2013.09.003 24070900

[B42] IntHout J. Ioannidis J. P. A. Borm G. F. (2014). The Hartung-Knapp-Sidik-Jonkman method for random effects meta-analysis is straightforward and considerably outperforms the standard DerSimonian-Laird method. BMC Med. Res. Methodol. 14:25. 10.1186/1471-2288-14-25 24548571 PMC4015721

[B43] Jagendorf A. T. Takabe T. (2001). Inducers of glycine betaine synthesis in barley. Plant Physiol. 127, 1827–1835. 10.1104/pp.010392 11743126 PMC133586

[B44] James D. W. Tindall T. A. Hurst C. J. Hussein N. (1995). Alfalfa cultivar responses to phosphorous and potassium deficiency: Biomass. J. Plant Nutr. 18, 2413–2445

[B45] Jayne B. Quigley M. (2014). Influence of arbuscular mycorrhiza on growth and reproductive response of plants under water deficit: a meta-analysis. Mycorrhiza 24, 109–119. 10.1007/s00572-013-0515-x 23917611

[B46] Jindal V. Atwal A. Sekhon B. S. Singh R. (1993). Effect of vesicular-arbuscular mycorrhizae on metabolism of moong plants under NaCl salinity. Plant Physiol. Biochem. 31, 475–481

[B47] Jones R. G. W. Gorham J. (1983). Osmoregulation. Physiol. Plant Ecol. 12, 33–58

[B48] Jung S. C. Martinez-Medina A. Lopez-Raez J. A. Pozo M. J. (2012). Mycorrhiza-induced resistance and priming of plant defenses. J. Chem. Ecol. 38, 651–664. 10.1007/s10886-012-0134-6 22623151

[B49] Kafkafi U. Gilat R. Yoles D. Noy Y. (1977). Studies on fertilization of field-grown irrigated alfalfa I. Effect of potassium source and time of application. Plant Soil 46, 165–173. 10.1007/BF00693123

[B50] Lajeunesse M. J. Forbes M. R. (2003). Variable reporting and quantitative reviews: a comparison of three meta-analytical techniques. Ecol. Lett. 6, 448–454. 10.1046/j.1461-0248.2003.00448.x

[B51] Lehmann A. Barto E. K. Powell J. R. Rillig M. C. (2012). Mycorrhizal responsiveness trends in annual crop plants and their wild relatives-a meta-analysis on studies from 1981 to 2010. Plant Soil 355, 231–250. 10.1007/s11104-011-1095-1

[B52] Leyva A. Quintana A. Sánchez M. Rodríguez E. N. Cremata J. Sánchez J. C. (2008). Rapid and sensitive anthrone–sulfuric acid assay in microplate format to quantify carbohydrate in biopharmaceutical products: Method development and validation. Biologicals 36, 134–141. 10.1016/j.biologicals.2007.09.001 18042399

[B53] Maya M. A. Matsubara Y. (2013). Influence of arbuscular mycorrhiza on the growth and antioxidative activity in cyclamen under heat stress. Mycorrhiza 23, 381–390. 10.1007/s00572-013-0477-z 23334657

[B54] Mayerhofer M. S. Kernaghan G. Harper K. A. (2013). The effects of fungal root endophytes on plant growth. Mycorrhiza 23, 199–128. 10.1007/s00572-012-0456-9 22983627

[B55] McGrath J. M. Lobell D. B. (2013). Reduction of transpiration and altered nutrient allocation contribute to nutrient decline of crops grown in elevated CO2 concentrations. Plant Cell Environ. 36, 697–705. 10.1111/pce.12007 22943419

[B56] Miransari M. (2013). Corn (*Zea mays* L.) growth as affected by soil compaction and arbuscular mycorrhizal fungi. J. Plant Nutr. 36, 1853–1867. 10.1080/01904167.2013.816729

[B57] Moftah A. E. Michel B. E. (1987). The effect of sodium chloride on solute potential and proline accumulation in soybean leaves. Plant Physiol. 83, 238–240. 10.1104/pp.83.2.238 16665227 PMC1056338

[B58] Morgan J. M. (1984). Osmoregulation and water stress in higher plants. Ann. Rev. Plant Physiol. 35, 299–319. 10.1146/annurev.pp.35.060184.001503

[B59] Munns R. James R. A. Läuchli A. (2006). Approaches to increasing the salt tolerance of wheat and other cereals. J. Exp. Bot. 57, 1025–1043. 10.1093/jxb/erj100 16510517

[B60] Nadeem S. M. Ahmad M. Zahir A. Javaid A. Ashraf M. (2014). The role of mycorrhizae and plant growth promoting rhizobacteria (PGPR) in improving crop productivity under stressful environments. Biotechnology Adv. 32, 429–448. 10.1016/j.biotechadv.2013.12.005 24380797

[B61] Pitman M. G. Läuchli A. (2002). Global impact of salinity and agricultural ecosystems, in Salinity: Environment – Plants – Molecules, ed Läuchli A. (Kluwer: Dordrecht), 3–20

[B62] Porcel R. Aroca R. Ruiz-Lozano J. M. (2012). Salinity stress alleviation using arbuscular mycorrhizal fungi. A review. Agron. Sust. Devel. 32, 181–200. 10.1007/s13593-011-0029-x

[B63] Porcel R. Ruíz-Lozano J. M. (2004). Arbuscular mycorrhizal influence on leaf water potential, solute accumulation, and oxidative stress in soybean plants subjected to drought stress. J. Exp. Bot. 55, 1743–1750. 10.1093/jxb/erh188 15208335

[B64] Querejeta J. I. Allen M. F. Caravaca F. A. Roldan A. (2006). Differential modulation of host plant δ^13^C and δ^18^O by native and nonnative arbuscular mycorrhizal fungi in a semiarid environment. New Phytol. 169, 379–387. 10.1111/j.1469-8137.2005.01599.x 16411940

[B65] Rai A. K. Takabe T. (2006). Abiotic Stress Tolerance in Plants: Toward the Improvement of Global Environment and Food. New York, NY: Springer

[B66] Rogatgi A. (2011). WebPlotDigitizer. Available online at: http://arohatgi.info/WebPlotDigitizer/app/ (Accessed March-June 2014).

[B67] Rosenberg M. S. Adams D. C. Gurevitch J. (2000). MetaWin: Statistical Software for Meta-Analysis, Version 2. Sunderland: Sinauer Associates

[B68] Rosendahl C. N. Rosendahl S. (1991). Influence of vesicular-arbuscular mycorrhizal fungi (*Glomus* spp.) on the response of cucumber (*Cucumis sativus* L.) to salt stress. Environ. Exp. Bot. 31, 313–318. 10.1016/0098-8472(91)90055-S

[B69] Ruíz-Lozano J. M. Aroca R. (2010a). Modulation of aquaporin genes by the arbuscular mycorrhizal symbiosis in relation to osmotic stress tolerance; in Symbioses and Stress, Vol. 17, *Cellular Origin, Life in Extreme Habitats and Astrobiology*, eds Seckbach J. Grube M. (Berlin: Springer), 357–374

[B70] Ruiz-Lozano J. M. Azcón R. (2000). Symbiotic efficiency and infectivity of an autochthonous arbuscular mycorrhizal *Glomus sp*. from saline soils and *Glomus deserticola* under salinity. Mycorrhiza 10, 137–143. 10.1007/s005720000075

[B71] Ruíz-Lozano J. M. Azcón R. Gómez M. (1996). Alleviation of salt stress by arbuscular-mycorrhizal Glomus species in *Lactuca sativa* plants. Physiol. Plant 98, 767–772. 10.1111/j.1399-3054.1996.tb06683.x

[B72] Ruiz-Lozano J. M. Gómez M. Azcón R. (1995). Influence of different *Glomus* species on the time-course of physiological plant responses of lettuce to progressive drought stress periods. Plant Sci. 110, 37–44. 10.1016/0168-9452(95)04184-V

[B73] Ruíz-Lozano J. M. Perálvarez M. C. Aroca R. Azcón R. (2011). The application of a treated sugar beet waste residue to soil modifies the responses of mycorrhizal and non mycorrhizal lettuce plants to drought stress. Plant Soil. 346, 153–166. 10.1007/s11104-011-0805-z

[B74] Ruíz-Lozano R. M. Aroca R. (2010b). Host Response to osmotic stresses – stomatal behaviour and water use efficiency of arbuscular mycorrhizal plants, in Arbuscular Mycorrhizas: Physiology and Function, eds Koltai H. Kapulnik Y Y. (Netherlands: Springer), 239–256

[B75] Ruíz-Lozano J. M. Porcel R. Azcón C. Aroca R. (2012). Regulation by arbuscular mycorrhizae of the integrated physiological response to salinity in plants: new challenges in physiological and molecular studies. J. Exp. Bot. 63, 695–709. 10.1093/jxb/ers126 22553287

[B76] Sannazzaro A. I. Echeverria M. Alberto E. O. Ruiz O. A. Menendez A. B. (2007). Modulation of polyamine balance in Lotus glaber by salinity and arbuscular mycorrhiza. Plant Physiol. Biochem. 45, 39–46. 10.1016/j.plaphy.2006.12.008 17303429

[B77] Scheloske S. Maetz M. Schneider T. Hildebrandt U. Bothe H. Povh B. (2004). Element distribution in mycorrhizal and nonmycorrhizal roots of the halophyte Aster tripolium determined by proton induced X-ray emission. Protoplasma 223, 183–189. 10.1007/s00709-003-0027-1 15221523

[B78] Seguel A. Cumming J. R. Klugh-Stewart K. Cornejo P. Borie F. (2013). The role of arbuscular mycorrhizas in decreasing aluminium phytotoxicity in acidic soils: a review. Mycorrhiza 23, 167–183. 10.1007/s00572-013-0479-x 23328806

[B79] Serraj R. Sinclair T. R. (2002). Osmolyte accumulation: can it really help increase crop yield under drought conditions? Plant Cell Environ. 25, 333–341. 10.1046/j.1365-3040.2002.00754.x 11841674

[B80] Sharifi M. Ghorbanli M. Ebrahimzadeh H. (2007). Improved growth of salinity-stressed soybean after inoculation with salt pre-treated mycorrhizal fungi. J. Plant Physiol. 164, 1144–1151. 10.1016/j.jplph.2006.06.016 16919369

[B81] Valdés-Santiago L. Ruiz-Herrera J. (2014). Stress and polyamine metabolism in fungi. Front. Chem. 1:42. 10.3389/fchem.2013.00042 24790970 PMC3982577

[B82] Veresoglou S. D. Menexes G. Rillig M. C. (2012). Do arbuscular mycorrhizal fungi affect the allometric partition of host plant biomass to shoots and roots? A meta-analysis of studies from 1990 to 2010. Mycorrhiza 22, 227–235. 10.1007/s00572-011-0398-7 21710352

[B83] Yancey P. H. (1994). Compatible and counteracting solutes, in Cellular and Molecular Physiology of Cell Volume Regulation, ed Strange K. (Boca Raton, FL: CRC Press), 81–109

[B84] Yokoi S. Bressan R. A. Hasegawa P. M. (2002). Salt Stress Tolerance of Plants. JIRCAS Working Report no. 23, 25-33. Available Online at: http://www.plantstress.com/articles/salinity_m/salinity_m_files/jircas.pdf [7/9/2014]

[B85] Zeng G. P. Zhang X. Liu H. L. Tan Y. Zhu L. J. (2011). Effect of AM fungi on salt tolerance of *Carthamus tinctorius* under salt stress. Plant Physiol. Comm. 47, 1064–1068

[B86] Zhu J. (2003). Regulation of ion homeostasis under salt stress. Curr. Opin. Plant Biol. 6, 441–445. 10.1016/S1369-5266(03)00085-2 12972044

[B87] Zhu X. C. Song F. B. Xu H. W. (2010). Influence of arbuscular mycorrhiza on lipid peroxidation and antioxidant enzyme activity of maize plants under temperature stress. Mycorrhiza 20, 325–332. 10.1007/s00572-009-0285-7 19936801

