# Supplementary material for: Arbuscular mycorrhizal symbiosis and osmotic adjustment in response to NaCl stress: a meta-analysis
Source: Front Plant Sci. 2014 Oct 17;5:562. doi: 10.3389/fpls.2014.00562 (PMC4201091; doi:10.3389/fpls.2014.00562)
Supplement: Supplementary file 2 [file SupplementaryMaterial2.PDF]

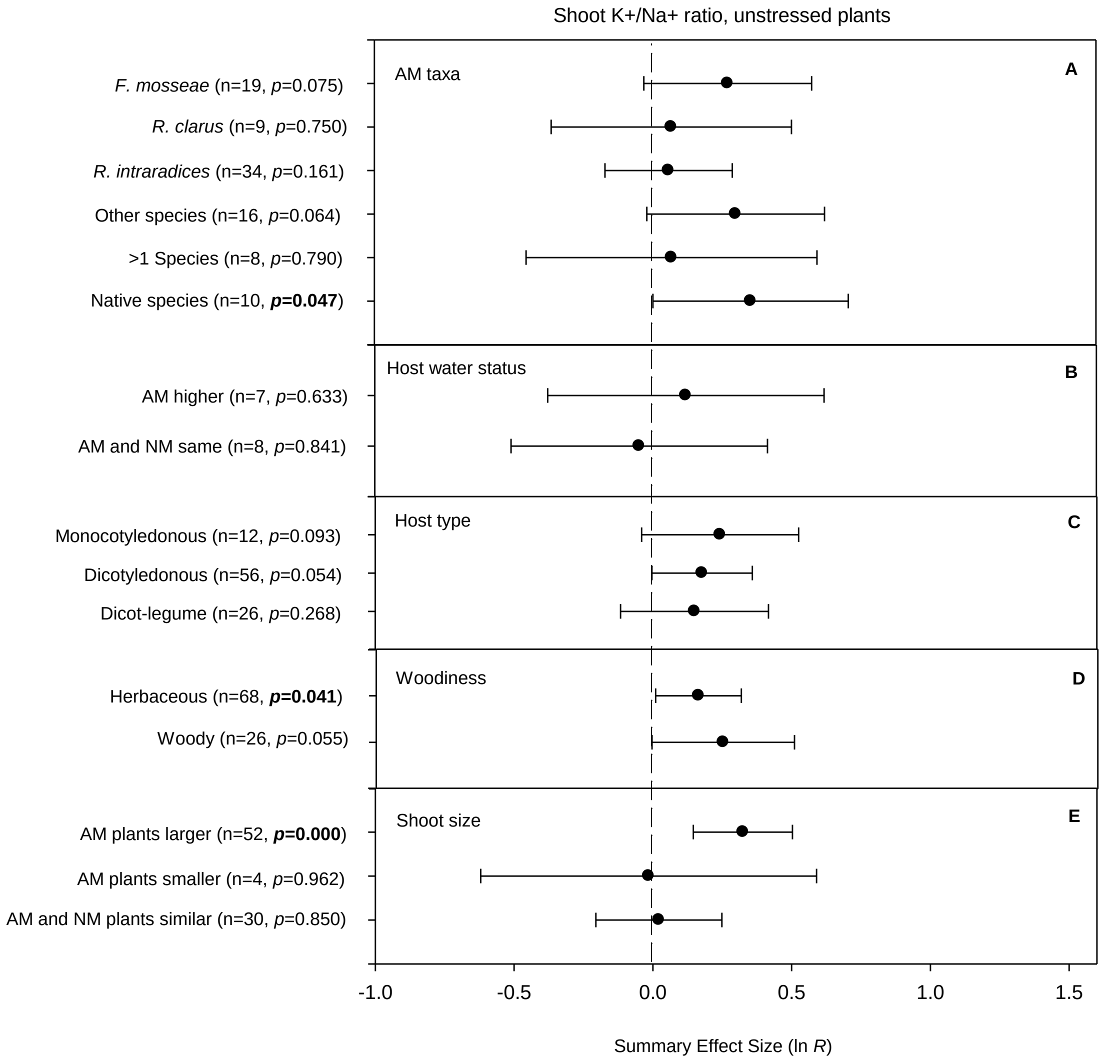

Figure 6. Weighted summary effect sizes (ln *R*) and 95% bootstrapped confidence intervals (CIs) for influence of AM mycorrhizal symbiosis on shoot K^+/Na^+ ratio in plants not exposed to NaCl stress (unstressed controls). Comparisons among moderator levels of (A) AM taxa, (B) Host water status, (C) Host type, (D) Woodiness, and (E) Shoot size. $p \leq 0.05$ indicates that the moderator level was significantly different than zero. Absent forest plots for a moderator or a particular level of a moderator reflects insufficient studies to have included that moderator or moderator level in the meta-analysis. A single fungal species was included as a level in the AM taxa moderator if at least 8 studies and >1 paper reported data for it. Those species having data from <8 studies or just 1 paper were grouped into "other species".

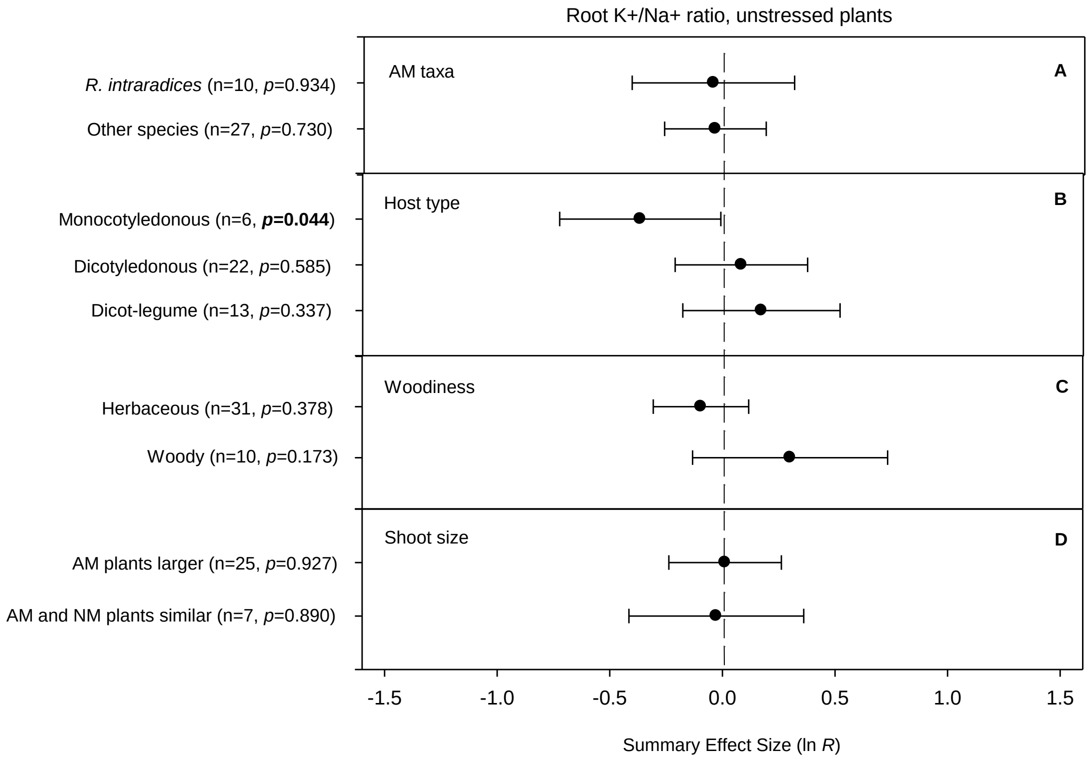

Figure 7. Weighted summary effect sizes (ln *R*) and 95% bootstrapped confidence intervals (CIs) for influence of AM mycorrhizal symbiosis on root K^+/Na^+ ratio in plants not exposed to NaCl stress (unstressed controls). Comparisons among levels of (A) AM taxa, (B) Host type, (C) Woodiness, and (D) Shoot size. $p \leq 0.05$ indicates that the moderator level was significantly different than zero. Absent forest plots for a moderator or a particular level of a moderator reflects insufficient studies to have included that moderator or moderator level in the meta-analysis. A single fungal species was included as a level in the AM taxa moderator if at least 8 studies and >1 paper reported data for it. Those species having data from <8 studies or just 1 paper were grouped into "other species".

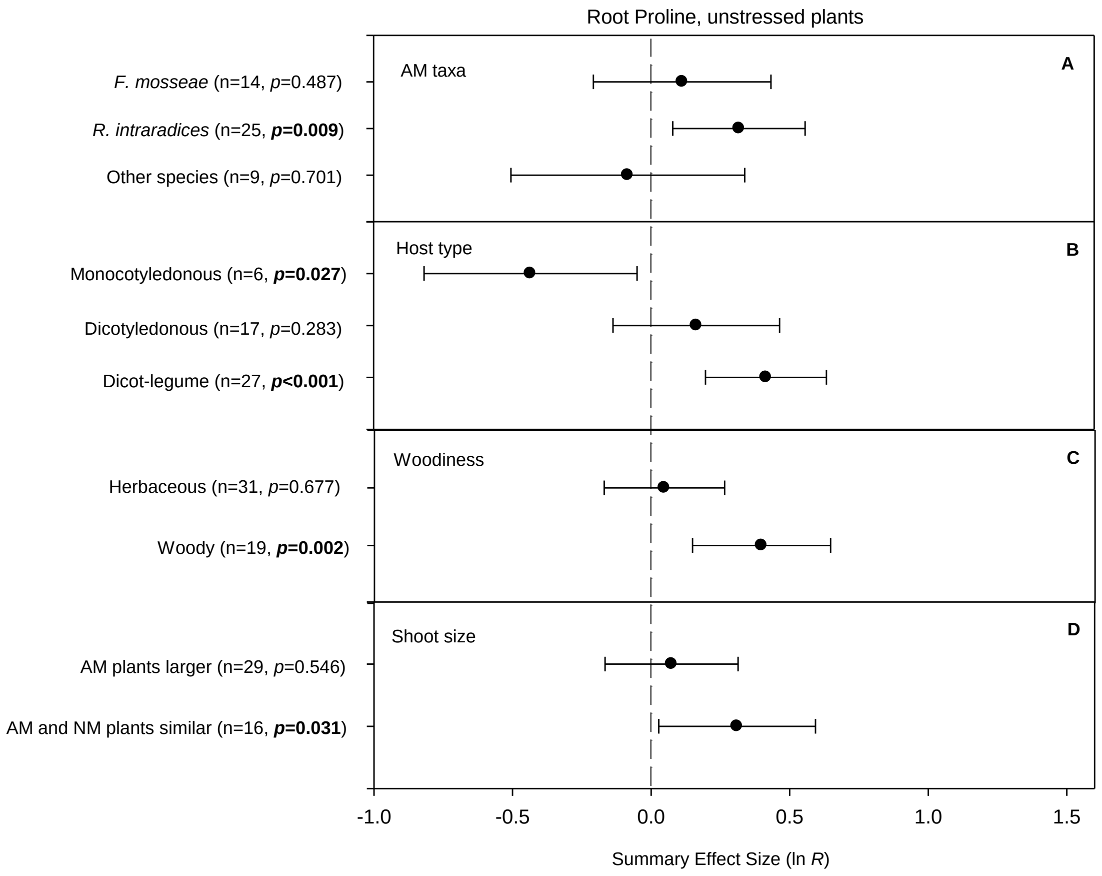

Figure 8. Weighted summary effect sizes (ln *R*) and 95% bootstrapped confidence intervals (CIs) for influence of AM mycorrhizal symbiosis on root proline in plants not exposed to NaCl stress (unstressed controls). Comparisons among levels of (A) AM taxa, (B) Host type, (C) Woodiness, and (D) Shoot size. $p \leq 0.05$ indicates that the moderator level was significantly different than zero. Absent forest plots for a moderator or a particular level of a moderator reflects insufficient studies to have included that moderator or moderator level in the meta-analysis. A single fungal species was included as a level in the AM taxa moderator if at least 8 studies and >1 paper reported data for it. Those species having data from <8 studies or just 1 paper were grouped into "other species".
